# Projecting the cost of introducing typhoid conjugate vaccine (TCV) in the national immunization program in Malawi using a standardized costing framework

**DOI:** 10.1016/j.vaccine.2022.02.016

**Published:** 2022-03-15

**Authors:** Frédéric Debellut, Rouden Mkisi, Vincent Masoo, Mike Chisema, Dennis Mwagomba, Mphatso Mtenje, Fumbani Limani, Donnie Mategula, Boston Zimba, Clint Pecenka

**Affiliations:** a Center for Vaccine Innovation and Access, PATH, Geneva, Switzerland; b Center for Vaccine Innovation and Access, PATH, Lilongwe, Malawi; c Health Management Information System, Mzuzu Central Hospital, Mzuzu, Malawi; d Expanded Programme on Immunization, Ministry of Health, Lilongwe, Malawi; e Malawi-Liverpool-Wellcome Trust/College of Medicine, Chichiri, Blantyre, Malawi; f Malawi-Liverpool-Wellcome Trust Clinical Research Programme, Queen Elizabeth Central Hospital Blantyre, Malawi; g World Health Organization, Lilongwe, Malawi; h Center for Vaccine Innovation and Access, PATH, Seattle, USA

**Keywords:** Typhoid conjugate vaccines, Immunization delivery cost, Malawi

## Abstract

**Background:**

There is a substantial typhoid burden in sub-Saharan Africa, and TCV has been introduced in two African countries to date. Decision-makers in Malawi decided to introduce TCV and applied for financial support from Gavi, the Vaccine Alliance in 2020. The current plan is to introduce TCV as part of the national immunization program in late 2022. The introduction will include a nationwide campaign targeting all children aged 9 months to 15 years. Following the campaign, TCV will be provided through routine immunization at 9 months. This study aims to estimate the cost of TCV introduction and recurrent delivery as part of the national immunization program.

**Methods:**

This costing analysis is conducted from the government's perspective and focuses on projecting the incremental cost of TCV introduction and delivery for Malawi’s existing immunization program before vaccine introduction. The study uses a costing tool developed by Levin & Morgan through a partnership between the International Vaccine Institute and the World Health Organization and leverages primary and secondary data collected through key informant interviews with representatives of the Malawi Expanded Programme on Immunization team at various levels.

**Results:**

The total financial and economic costs of TCV introduction over three years in Malawi are projected to be US$8.5 million and US$29.8 million, respectively. More than two-thirds of the total cost is made up of recurrent costs. Major cost drivers include the procurement of vaccines and injection supplies and service delivery costs. Without vaccine cost, we estimate the cost per child immunized to be substantially lower than US$1.

**Discussion:**

Findings from this analysis may be used to assess the economic implications of introducing TCV in Malawi. Major cost drivers highlighted by the analysis may also inform decision-makers in the region as they assess the value and feasibility of TCV introduction in their national immunization program.

## 1 Introduction

Typhoid fever was responsible for more than 9 million infections and 110,000 deaths globally in 2019 [1]. In sub–Saharan Africa, the burden of typhoid is believed to have been long underestimated. Prior studies have estimated that typhoid may be responsible for more than 3 million cases and 33,000 deaths every year in the region [2]. More recent work reports incidence rates as high as 383 per 100,000 person-years in some African countries [3]. The increased frequency of reported typhoid outbreaks and the presence of antimicrobial-resistant typhoid on the African continent call for controlling efforts of the disease [4], [5].

In 2018, the World Health Organization (WHO) recommended introducing typhoid conjugate vaccine (TCV) in typhoid-endemic settings [6]. Despite the challenge posed by typhoid in sub-Saharan Africa, TCV was only introduced in two African countries by the end of 2021 [7]. There are currently two vaccines available through support from Gavi. TYPBAR TCV (Bharat Biotech International, India) and TYPHIBEV (Biological Evans Limited, India) are both available in 5-dose vial liquid presentation [8].

Disease burden is an essential driver of vaccine introduction decisions. Typhoid fever surveillance has been implemented for more than two decades in Malawi, and more recently, a clinical trial of TCV effectiveness was completed [9], [10]. Typhoid fever incidence in Malawi has risen in recent years, likely driven by drug resistance [11], [12]. Recent data show that more than 90% of *Salmonella* Typhi isolates in Blantyre area were multi-drug resistant [13]. It is estimated that in 2017, Malawi experienced 32,747 typhoid cases (an incidence of 191 cases per 100,000 population—61% of which were among children under 15 years old), and 435 typhoid deaths (66% of which were among children under 15 years old) [14]. Vaccine program costs are also an essential factor in vaccine introduction decisions. There are currently few data on TCV program costs, a gap this analysis helps fill.

Policymakers in Malawi decided to introduce TCV and applied for financial support through Gavi, the Vaccine Alliance (Gavi), in September 2020. The current plan is to introduce TCV as part of the national immunization program in late 2022. The introduction will include a nationwide campaign targeting all children aged 9 months to 15 years old. Following the campaign, TCV will be provided through routine immunization at 9 months of age. This study aims to estimate the cost of TCV introduction (startup cost) and delivery (recurrent cost) as part of the national immunization program. Although TCV will be delivered as part of the routine immunization program, the strategy requires a combination of routine and campaign deliveries targeting different populations. Given that these may differ from other new vaccines introduced in the past, the delivery cost may be different. Understanding the costs of introducing TCV in Malawi is integral to evaluating the programmatic feasibility of TCV introduction, informing budget planning, and ensuring sustained delivery. Findings from this study may inform decision-makers in Malawi and other countries interested in TCV introduction.

## 2 Methods

### 2.1 Study objectives and design

The primary objective of the costing study is to project the cost of TCV introduction (startup cost) and delivery (recurrent cost) in Malawi in the context of both routine and campaign delivery over three years, starting in 2022. We decided to project costs over three years to account for all activities related to the introduction, with a large campaign in the first year and two follow on years of routine delivery of the vaccine. A secondary objective is to determine the significant cost drivers of the TCV introduction and cost per child immunized.

The study follows an activity-based costing approach, where all activities associated with the introduction and delivery of the vaccine are identified, measured, and valued individually. This costing analysis takes the government’s perspective and focuses on the incremental cost of adding TCV to the existing immunization program. Both financial and economic costs are included in the analysis. While financial costs are restricted to direct expenses or monetary outlays, economic costs provide a broader perspective to account for the opportunity cost of resources that would have otherwise been allocated to other activities and programs [15].

The costing analysis gathered and utilized data on activities, costs/expenditures, resources, and outcomes (mostly coverage assumptions) obtained through key informant interviews of Expanded Programme on Immunization (EPI) personnel working on immunization program management at different health system levels. We followed the standard guidelines in estimating the costs of TCV introduction and delivery [15], [16], [17]. The key outputs of the analysis include the financial and economic costs per fully immunized child (or per dose delivered, since TCV requires a single dose for a full course of vaccination), the cost drivers of vaccine introduction and delivery, and the total financial and economic cost of TCV introduction and delivery in Malawi over three years. All cost data (besides vaccine and supplies procurement) were collected and entered into a costing tool in local currency and further converted to US$ using an exchange rate of MWK812.9 for US$1 (www.xe.com September 7, 2021). To combine them with recurrent costs, we annualized introduction costs, assuming they would have a useful life year of three years (and using a 3% discount rate for economic costs).

### 2.2 TCV costing tool

Cost estimates per cost category per fully immunized children and the total cost over three years were projected using a Microsoft Excel-based costing tool. This TCV costing tool—jointly developed by the WHO, IVI, and Levin & Morgan—was developed to facilitate decision-making by providing program managers and policymakers with data on the projected cost of introduction. It provides a standardized framework available to governments to generate TCV immunization program cost data by estimating the value of incremental resources required to introduce TCV into an existing national immunization program and deliver the vaccine over a given period. The costing tool builds on previously developed tools such as the WHO Cervical Cancer Prevention and Control Costing (C4P) tool or the Seasonal Influenza Immunization Costing Tool (SIICT, Flutool Plus). These and other tools have been used in other settings for similar analyses [18], [19], [20], [21], [22], [23]. The TCV costing tool is comprised of a series of Excel sheets in which users enter unit costs and quantities of resources used for a list of major activities, with each Excel worksheet corresponding to a major activity. The list of activities and classification between introduction and recurrent cost categories included in the tool are shown in supplementary Table S1.

### 2.3 Study area, sampling, and data collection

We collected data from the national immunization program at the central level and a selection of four districts and six health facilities within each of the selected districts. Districts and health facilities were purposively selected in collaboration with the EPI team, based on several criteria reflecting a variety of geographies (urban/rural/hard-to-reach), population densities (high/medium/low), and accounting for variable EPI performance using Pentavalent 1 and Pentavalent 3 coverage rates as an indicator, covering most characteristics encountered in the country. Selected districts represent more than 28% of the country target population. We selected one district with a high population and high volume of activity, one district with low immunization performance, one district where immunization delivery is undertaken using a multiplicity of transportation means due to its geography, and one district with long distances between facilities and low population density. Districts included in the analysis are available in Fig S1, criteria for selection are available in Table S2.

Data collection took place at the National Immunization Program and the National Vaccine Store at the national level*.* Key informants included the National Logistics and Supply Chain Officer and the National Data Manager. In the four districts, the district EPI officer and the district Vaccine Store Manager were interviewed. Data were collected using forms developed to capture all expenses and resources (unit cost and quantities) associated with implementing a list of activities and sub-activities identified by key informants at the time of the interview. The list of activities and sub-activities identified are available in supplementary Table S3. No activities or additional resources were identified for categories such as additional cold chain equipment or adverse event following immunization (AEFI).

We extrapolated cost to all districts, assuming an average cost for each sub-activity. Because Lilongwe district is quite different from the rest of the country, with a much larger population and the biggest urban area of the country, we applied costs collected in this district to Lilongwe only and used an average of the cost collected in the three other districts to others. Because of the uncertainty in cost at district level outside of the districts where data collection took place, we also ran an additional scenario using the average across the four districts visited. We will report these results as sensitivity analysis.

At the health facility level, key informants were the health facility EPI focal point and the health facility senior health surveillance assistant (supervisor of other health surveillance assistants). In health facilities, standard costing questionnaires were used to collect data on resources and time spent on routine immunization activities for static, outreach, and mobile clinics’ delivery modes. For example, resources included cost for per diem, transportation, or fuel. Time spent by facility staff involved in providing immunization services, managing vaccine stock, program management and monitoring, and waste management was also collected. In addition, data on doses administered and wasted for all antigens in the immunization system in 2019 were also recorded from each health facility visited. Data collection focused on 2019 as a reference year for projections rather than 2020 due to the disruptions experienced during the onset of the COVID-19 pandemic. Data on the number of doses used annually were used to calculate a cost per dose delivered in the routine system that was applied to the population covered by the TCV program in each district.

Time of immunization program staff participating in the different activities was valued based on the national salary scale [24]. Volunteer time was valued using the minimum wage in Malawi (Ministry of Labour Act of January 2021 was MWK 50,000.00 per month). The analysis did not account for time spent on activities by staff from international organizations or stakeholders outside of government staff.

Expected coverage rates for the campaign and routine strategies were collected from the national EPI team. Based on their information, we assumed that an initial campaign would occur in 2022, targeting children from 9 months to under 15 years old in the entire country, with an expected coverage of 95%. In follow-on years, routine immunization will target 9-month-olds only, with an expected coverage rate of 80% in 2023, increasing to 84% in 2024, in line with coverage levels of the first dose of measles-containing-vaccine (MCV1) given at the same age in Malawi. [25] Population data for the two target populations were taken from the National Statistics Office [26]. Because of the uncertainty around coverage rates that may be achieved at the time of introduction, we explored several scenarios accounting for lower coverage rates (from 10 to 30 percentage point reduction).

Vaccine-related costs were gathered from Gavi, as well as the UNICEF country office [8]. For the campaign, the cost of vaccines and supplies are covered by Gavi. For routine immunization, Malawi pays a co-financing of $0.20 per dose of vaccine as an initial self-financing country and per Gavi’s co-financing policy [27], [28], [29]. Economic costs do account for the full price of vaccine and supply procurement. The EPI logistics and local UNICEF office informed on additional costs of procurement such as freight, handling, insurance, and other costs. All vaccine-related costs and other key data inputs are listed in Table 1.

**Table 1:** Key data inputs, base scenario.

**Input**	**Value**	**Source**
**Target population**		
Campaign (2022)	8,901,871	[26]
Routine (2023)	812,719	
Routine (2024)	831,797	
**Doses per fully immunized child**	1	[6]
**Vaccine coverage**		
Campaign (2022)	95%	Assumption from central EPI
Routine (2023)	80%	
Routine (2024)	84%	
**Vaccine price**		
Campaign	$0 financial cost, $1.50 economic cost	[27], [28], [29]
Routine	$0.20 financial cost, $1.50 economic cost	
**Vaccine presentation**	5-dose vial liquid	[8]
**Vaccine wastage rate**	10%	Assumption
**Vaccine procurement additional costs** (% of vaccine price)	21%	UNICEF country office
**Syringe price**	$0.05	
**Syringe wastage rate**	10%	Assumption
**Safety box price** (100 injection syringes capacity)	$0.50	UNICEF country office
**Safety box wastage rate**	5%	Assumption
**Supplies procurement additional costs** (% of supply price)	31%	UNICEF country office

We report total financial and economic costs, per category and annually. We also report an overall cost per fully immunized child vaccinated with TCV. We report a similar ratio, differentiating the cost to immunize children as part of the campaign and as part of the routine immunization system. To differentiate costs between the campaign and routine settings, we used the respective target population of each delivery strategy to distribute introduction costs. We further separated vaccine procurement and service delivery costs based on the population covered by each strategy in the program. As we applied an incremental costing methodology, we did not collect costs of immunization-related activities integrated in the routine system. For example, routine supportive supervision, social mobilization, IEC materials, or other costs were not included.

### 2.4 Ethical considerations

The PATH Research Determination Committee reviewed this study and determined it did not involve the use of human subjects nor represent research (RES-00119). In Malawi, the study protocol received ethical approval from the National Health Sciences Research Committee (Protocol # 21/05/2890).

## 3 Results

We estimate the total financial cost of TCV introduction over three years in Malawi to be US$8.6 million and the economic cost US$29.8 million (Table 2). This total cost is largely attributable to recurrent costs, with a proportion of 77.5% and 86.1%, respectively, for the financial and economic costs. From a financial cost perspective, major cost drivers include the procurement of vaccines and injection supplies (44.6%), followed by service delivery costs (18.2%), training (10.9%), microplanning (10.6%), and social mobilization and communications (9.3%).

**Table 2:** Projected total financial and economic costs of TCV immunization program (not annualized).

Activity	Financial Costs 2022–2024, USD (% of total)	Economic Costs 2022–2024, USD (% of total)
**Introduction costs**	**$1,922,024 (22.5%)**	**$4,131,689 (13.9%)**
Program planning and preparation	$63,100 (0.7%)	$85,710 (0.3%)
Microplanning	$902,205 (10.6%)	$2,272,088 (7.6%)
Training	$934,572 (10.9%)	$1,745,216 (5.9%)
Sensitization	$22,146 (0.3%)	$28,674 (0.1%)
**Recurrent**	**$6,617,838 (77.5%)**	**$25,683,281 (86.1%)**
Vaccine and injection supply procurement	$3,980,344 (46.6%)	$20,419,775 (68.5%)
Social mobilization and communication	$795,852 (9.3%)	$1,239,909 (4.2%)
Service delivery costs	$1,555,807 (18.2%)	$3,423,071 (11.5%)
Supervision and monitoring	$211,456 (2.5%)	$429,296 (1.4%)
Other recurrent costs	$74,378 (0.9%)	$171,230 (0.6%)
**Total Costs**	**$8,539,861 (100.0%)**	**$29,814,969 (100.0%)**

From 2022 to 2024, a total of 9.8 million children younger than 15 years old are projected to be vaccinated with one dose of TCV, including 8.5 million children during the initial campaign in 2022. With our assumptions, the financial cost to immunize a child with TCV in Malawi is US$0.87 when accounting for vaccine cost. Without vaccine costs, the total is US$0.46 (Table 3). The economic cost to immunize a child is US$3.03 with vaccine cost, or US$0.96 without vaccine cost. When differentiating costs for the campaign from the routine system, we came up with a financial cost of US$0.86 per child immunized in the campaign and US$0.92 per child immunized in the routine immunization system, vaccine cost included. When excluding vaccine cost, the cost to immunize children in the routine immunization system is lower than during the campaign, with a financial cost per child of US$0.27 compared to US$0.50 in routine immunization. The annual financial cost projected (Table 4) reflects our assumption of the campaign occurring in 2022, followed by routine immunization in 2023 and 2024, with a higher cost in the introduction year (US$6.3 million) than in follow-on years (US$1.1 million each subsequent year).

**Table 3:** Cost per fully immunized child.

	**Overall TCV program**		**Campaign**		**Routine**	
	**Financial cost per child**	**Economic cost per child**	**Financial cost per child**	**Economic cost per child**	**Financial cost per child**	**Economic cost per child**
**With vaccine cost**	$0.87	$3.03	$0.86	$3.06	$0.92	$2.83
**Without vaccine cost**	$0.46	$0.96	$0.50	$0.99	$0.27	$0.76

**Table 4:** Annualized projected total financial costs of TCV immunization program.

Activity	2022	2023	2024
**Introduction costs**	**$640,674**	**$640,675**	**$640,675**
Program planning and preparation	$21,033	$21,033	$21,033
Microplanning	$300,735	$300,735	$300,735
Training	$311,524	$311,524	$311,524
Sensitization	$7,382	$7,382	$7,382
**Recurrent**	**$5,658,658**	**$462,350**	**$496,829**
Vaccine and injection supply procurement	$3,088,880	$429,710	$461,754
Social mobilization and communication	$795,852	$0	$0
Service delivery costs	$1,488,092	$32,641	$35,075
Supervision and monitoring	$211,456	$0	$0
Other recurrent costs	$74,318	$0	$0
**Total Costs**	**$6,299,332**	**$1,103,025**	**$1,137,504**

When we account for an average cost across all four districts instead of treating the Lilongwe district separately (Table S4), the financial cost increases by 15.7% or US$1.3 million. This does not change the main cost drivers, however. This change mainly impacts introduction costs with an increase of 46.5% compared to recurrent costs which increase by only 6.7%. The larger increase in startup costs is driven by the higher training and microplanning costs in Lilongwe districts.

When we account for lower coverage rates, we see a reduction in total financial and economic costs. A 10 percentage point reduction in coverage rate translates in a 5% reduction of the total financial cost. A 30 percentage point reduction in coverage rate translates in a 15% reduction of the total financial cost (Table S5).

## 4 Discussion

Our study projects the financial cost of TCV introduction over three years in Malawi to be US$8.5 million and the economic cost to be US$29.8 million. Main cost drivers include procurement of the vaccine and injection supplies and service delivery. The financial cost to immunize a child with TCV is estimated to be less than US$1. The financial cost projected for TCV introduction represents 4% of the total estimated budget required for the last 5-year planning for all immunization activities in Malawi [30].

This analysis accounts for the full support provided by Gavi on vaccine and injection supplies procurement during the initial campaign. The government of Malawi pays only a share of the vaccine price based on the Gavi co-financing policy for vaccines used in the routine immunization system [27], [28], [29]. It should be noted that even with vaccine and injection supplies procurement fully supported by Gavi, there remains a cost to the government related to the freight, handling, insurance, and other costs on commodities. The high number of doses required for the campaign makes that cost substantial in the first year of introduction.

There is additional financial support available to Malawi that is excluded from our analysis which may alleviate some of the TCV introduction costs. For the initial campaign, for a country in the initial self-financing phase such as Malawi, Gavi provides a Campaign Operational Support Grant of US$0.65 per child targeted. For the introduction in routine immunization, Gavi provides a Vaccine Introduction Grant equal to US$0.80 per child targeted for countries in the same transition phase [29]. These cash grants could substantially offset some of the costs related to TCV introduction, bringing the total financial cost down by as much as US$6.4 million. An additional way to reduce the cost of a TCV campaign that has been discussed in Malawi is the integration of the TCV campaign with a measles campaign, partially targeting the same population cohort. However, because of the timing of the study completion, we have not assessed how such integration may impact the overall cost of TCV introduction.

The incremental cost approach taken may have resulted in underestimating some of the recurrent costs required in follow-on years. As part of routine immunization, one may consider that a share of some cost categories could be allocated to TCV. For example, opportunity costs for supportive supervision, social mobilization activities, and other supply chain-related costs could be considered in the analysis even if they are integrated into the routine immunization system. Our study shows that the cost per child, without vaccine cost, is lower in the routine immunization system compared to the campaign. This may be surprising as we might expect economies of scale to be achieved with a campaign targeting a large population. However, the incremental approach may be one of the explanatory factors for the rather small cost of delivery in the routine system. Trying to capture all opportunity costs in the routine immunization system wasn’t feasible as this substantially extends the scope of data collection beyond incremental costs. More expansive studies of the full immunization program costs are better suited to answer this question.

This study projects costs based on assumptions and data collected from key informant interviews and from a few purposively sampled districts. As a result, our findings are not fully representative of costs in every district or nationwide. The cost projections we present in this paper should be confirmed by a further study leveraging similar methods but focusing on retrospective costing after TCV introduction. Our sensitivity analysis shows a potential increase in cost, which also argues in favor of a larger sample of districts and facilities to lead to more robust estimates, particularly to capture the extent of startup costs at district levels which sees the largest variation in expenses.

Although data collection took place almost a year into the COVID-19 pandemic, it remains unclear if our estimates fully account for additional costs related to COVID-19 preventative measures. One would expect some variations in the inclusion of these costs given information is based on various key informants. Most informants relied on their experience with a measles-rubella campaign in 2017, well before the COVID-19 preventative measures were included. The available research on the potential additional cost related to COVID-19 preventative measures has shown that it may substantially increase campaign and routine immunization delivery costs depending on the kind of preventative measures observed [31]. The estimates presented in this analysis should therefore be used with caution. The extent of implications on cost to introduce TCV will be dependent on the evolution of the COVID-19 pandemic. One may want to consider the situation at the time of introduction to assess how reflective or not they are of actual costs. Impact of COVID-19 preventative measures may be more easily captured with retrospective cost analysis, considering that they tend to fluctuate and are dependent on a series of factors—including availability of prevention commodities such as masks and sanitizers, disease perception, level of disease transmission, etc.

These cost projections may be used by the government of Malawi for budgeting purposes and to inform TCV delivery sustainability plans. Cost per dose delivered is also a key input for economic evaluation and may inform future cost-effectiveness studies. Policymakers in other countries in the region may use the findings of this study to inform their thinking about TCV introduction.

## Data availability statement

The authors confirm that the data supporting the findings of this study are available within the article and its supplementary materials.

## Declaration of Competing Interest

The authors declare that they have no known competing financial interests or personal relationships that could have appeared to influence the work reported in this paper: [Clint Pecenka reports financial support was provided by Bill and Melinda Gates Foundation.].
