# Supplementary material for: Projecting the cost of introducing typhoid conjugate vaccine (TCV) in the national immunization program in Malawi using a standardized costing framework
Source: Vaccine. 2022 Mar 15;40(12):1741–6. doi: 10.1016/j.vaccine.2022.02.016 (PMC8917043; doi:10.1016/j.vaccine.2022.02.016)
Supplement: Supplementary data 1 [file mmc1.docx]

**Supplementary files**

*Table S1. Major activities included in the TCV costing tool.*

| **Startup (Introduction) Costs** | **Recurrent (Ongoing) Costs** |
| --- | --- |
| - Program Planning & Preparation | - Vaccine and Injection Supply Procurement |
| - Microplanning | - Social Mobilization and Communication |
| - Training | - Service Delivery Costs |
| - Sensitization | - Supervision and Monitoring |
|  | - Adverse Events Following Immunization (AEFI) |
|  | - Other Activities |

Figure S1. Map of Malawi and districts included in the study.


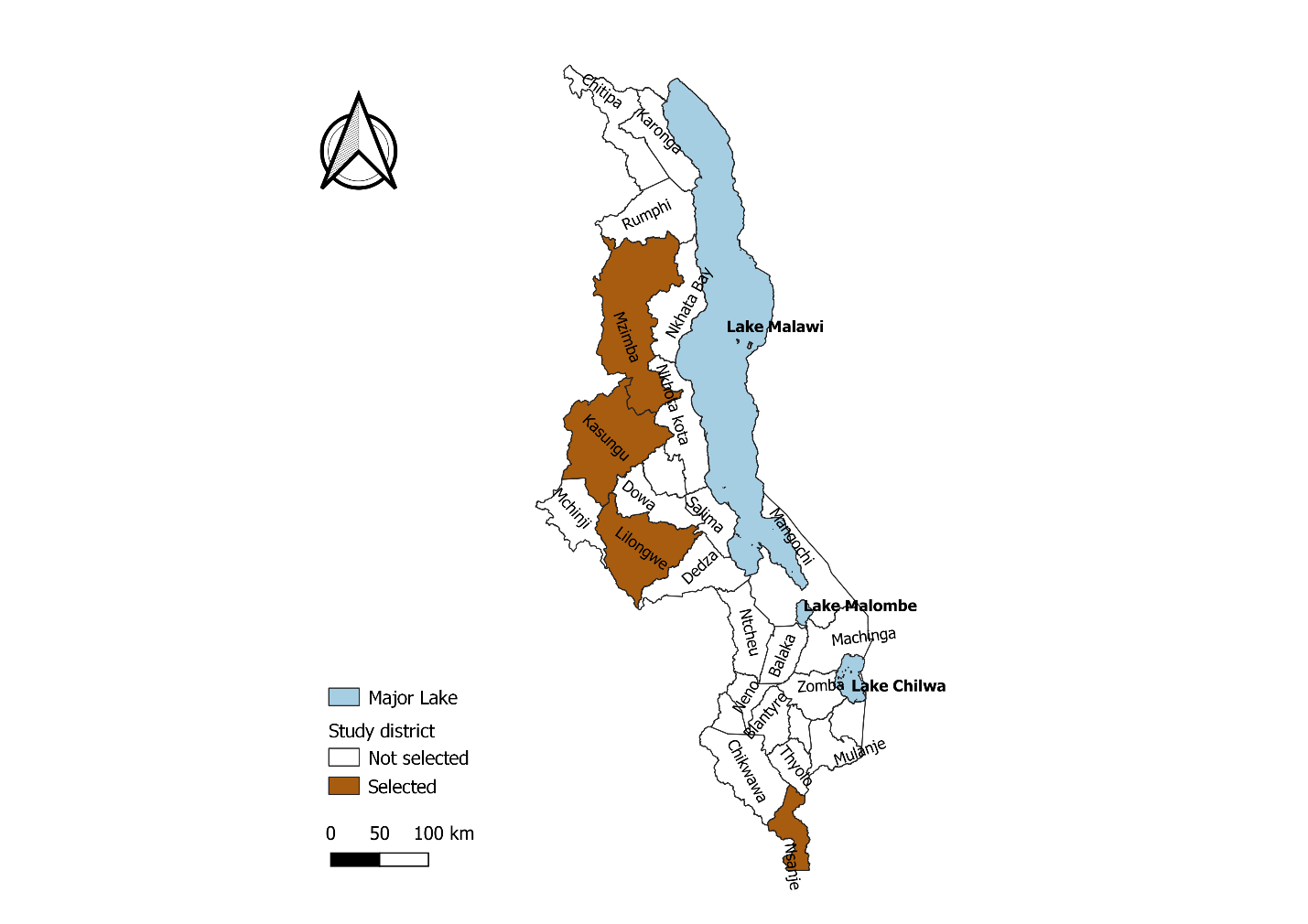


*Table S2. Districts and health facilities selected and selection criteria*

| **Districts’ selection** | | | | |  |
| --- | --- | --- | --- | --- | --- |
| **Criteria** | High volume district, high population density | Low immunization performance | Geography (immunization delivery using multiple means of transportation: boats, bicycles and motorized vehicles) | Geography – (long distances between health facilities, low population density) |  |
| **District name** | Lilongwe district | Kasungu district | Nsanje district | Mzimba South district |  |
|  |  |  |  |  |  |
| **Health facilities’ selection** | | | | | |
| **Criteria** | Urban facility | Low EPI performance | High EPI performance | Hard to reach facility | *District* |
| **Health facility name** | Area 18 health facility  Kawale health facility | Matapila health facility  Mitundu health facility | Bwaila health facility | Chimbalanga health facility | *Lilongwe district* |
|  | Mtunthama health facility  Kasungu District health facility | Mkhota health facility  Chulu health facility | Kasalika health facility | Simulemba health facility | *Kasungu district* |
|  | Trinity health facility  Nsanje District health facility | Masenjere health facility  Makhanga health facility | Sankhulani health facility | Chididi health facility | *Nsanje district* |
|  | Mzimba District health facility  Embangweni health facility | Edingeni health facility  Mzalangwe health facility | Luwawa health facility | Khosolo health facility | *Mzimba South district* |

*Table S3. Identified activities and sub-activities.*

| **National level activities and sub-activities** | **District level activities and sub-activities** |
| --- | --- |
| **Planning and preparation** | |
| National Task Force meeting | **-** |
| Readiness Assessment (data collection) | **-** |
| **Microplanning** | |
| Training district teams on methods for compiling health facility and district micro-plans | District-level microplanning and data collection |
| Consolidation of district micro-plans into national micro-plan | District Taskforce meeting |
| **Training** | |
| Review of TCV training materials | Training of district task force and district supervisors* |
| Training of trainers for all districts | Training of health workers |
| Briefing of the Ministry of Education’s senior management | Training of Trainers |
| Briefing of district task force teams on TCV campaign implementation | Training of volunteers* |
| **Sensitization** | |
| Publicity during TCV vaccine launch | **-** |
| Advance party for the TCV launch | **-** |
| Events on actual day of the launch | **-** |
| **Social mobilization and communications** | |
| Development of risk and communication strategy | Engagement of community radio |
| Development of messages and IEC materials for TCV (including pretesting and review) | Community mobilization |
| Briefing of media personnel on TCV campaign and introduction | Briefing of stakeholders |
| Production of radio and TV commercials | Community sensitization |
| **Service delivery** | |
| Distribution of vaccines and injection supplies to regional vaccine stores and further to district vaccine storerooms | Distribution of vaccines |
| **-** | Administration of TCV vaccines during the campaign’s static and outreach sessions |
| **-** | Mop-up activities |
| **-** | Routine administration of TCV |
| **Supervision and monitoring** | |
| Supervision during training of health workers | Pre-campaign support supervision |
| Pre-campaign support supervision | District-level supportive supervision |
| Supervision during actual campaign days | **-** |
| Supervision during mop up campaign days | **-** |
| Review of monitoring and reporting tools | **-** |
| **Other recurrent costs** | |
| TCV campaign report writing | Waste management–safe disposal |

* Lilongwe district only

*Table S4. Comparison of financial and economic costs, base case scenario vs. alternative scenario.*

|  | **Lilongwe and three other districts average**  Base case scenario | | **Four districts average**  Alternative scenario | |
| --- | --- | --- | --- | --- |
| Activity | Financial Costs  2022-2024, USD (% of total) | Economic Costs 2022-2024, USD (% of total) | Financial Costs 2022-2024, USD (% of total) | Economic Costs 2022-2024, USD (% of total) |
| **Introduction costs** | **$1,922,024 (22.5%)** | **$4,131,689 (13.9%)** | **$2,815,342 (28.5%)** | **$6,080,206 (18.5%)** |
| Program planning and preparation | $63,100 (0.7%) | $85,710 (0.3%) | $63,100 (0.6%) | $85,710 (0.3%) |
| Microplanning | $902,205 (10.6%) | $2,272,088 (7.6%) | $1,220,421 (12.4%) | $3,181,073 (9.7%) |
| Training | $934,572 (10.9%) | $1,745,216 (5.9%) | $1,509,675 (15.3%) | $2,784,749 (8.5%) |
| Sensitization | $22,146 (0.3%) | $28,674 (0.1%) | $22,146 (0.2%) | $28,674 (0.1%) |
| **Recurrent** | **$6,617,838 (77.5%)** | **$25,683,281 (86.1%)** | **$7,061,505 (71.5%)** | **$26,748,445 (81.5%)** |
| Vaccine and injection supply procurement | $3,980,344 (46.6%) | $20,419,775 (68.5%) | $3,980,344 (40.3%) | $20,419,775 (62.2%) |
| Social mobilization and communication | $795,852 (9.3%) | $1,239,909 (4.2%) | $798,065 (8.1%) | $1,235,366 (3.8%) |
| Service delivery costs | $1,555,807 (18.2%) | $3,423,071 (11.5%) | $2,022,990 (20.5%) | $4,516,932 (13.8%) |
| Supervision and monitoring | $211,456 (2.5%) | $429,296 (1.4%) | $194,467 (2.0%) | $427,864 (1.3%) |
| Other recurrent costs | $74,378 (0.9%) | $171,230 (0.6%) | $65,639 (0.7%) | $148,509 (0.5%) |
| **Total Costs** | **$8,539,861 (100.0%)** | **$29,814,969 (100.0%)** | **$9,876,848 (100.0%)** | **$32,828,650 (100.0%)** |

Table S5 Comparison of total financial and economic costs, base case scenario vs. lower coverage scenarios

|  | **Total financial cost** | **Percentage reduction compared to base case** | **Total economic cost** | **Percentage reduction compared to base case** |
| --- | --- | --- | --- | --- |
| **Base case scenario** | $8,539,861 | - | $29,814,969 | - |
| **10 percentage points coverage reduction**  *Vaccine coverage*  *Campaign (2022) = 85%*  *Routine (2023) = 70%*  *Routine (2024) = 74%* | $8,097,776 | 5% | $27,570,374 | 8% |
| **20 percentage points coverage reduction**  *Vaccine coverage*  *Campaign (2022) = 75%*  *Routine (2023) = 60%*  *Routine (2024) = 64%* | $7,655,690 | 10% | $25,325,779 | 15% |
| **30 percentage points coverage reduction**  *Vaccine coverage*  *Campaign (2022) = 65%*  *Routine (2023) = 50%*  *Routine (2024) = 54%* | $7,228,506 | 15% | $23,131,744 | 20% |
